# The Emerging Regulatory Role of Circular RNAs in Periodontal Tissues and Cells

**DOI:** 10.3390/ijms22094636

**Published:** 2021-04-28

**Authors:** Kexin Jiao, Laurence J. Walsh, Sašo Ivanovski, Pingping Han

**Affiliations:** 1 Epigenetics Nanodiagnostic and Therapeutic Group, Center for Oral-Facial Regeneration, Rehabilitation and Reconstruction (COR3), School of Dentistry, Faculty of Health and Behavioural Sciences, The University of Queensland, Brisbane, QLD 4006, Australia; kexin.jiao@uq.net.au; 2 School of Dentistry, Faculty of Health and Behavioural Sciences, The University of Queensland, Brisbane, QLD 4006, Australia; l.walsh@uq.edu.au

**Keywords:** circular RNAs, epigenetics, pathogenesis, biomarkers, periodontal regeneration, osteogenic differentiation

## Abstract

Periodontitis is a chronic complex inflammatory disease associated with a destructive host immune response to microbial dysbiosis, leading to irreversible loss of tooth-supporting tissues. Regeneration of functional periodontal soft (periodontal ligament and gingiva) and hard tissue components (cementum and alveolar bone) to replace lost tissues is the ultimate goal of periodontal treatment, but clinically predictable treatments are lacking. Similarly, the identification of biomarkers that can be used to accurately diagnose periodontitis activity is lacking. A relatively novel category of molecules found in oral tissue, circular RNAs (circRNAs) are single-stranded endogenous, long, non-coding RNA molecules, with covalently circular-closed structures without a 5’ cap and a 3’ tail via non-classic backsplicing. Emerging research indicates that circRNAs are tissue and disease-specific expressed and have crucial regulatory functions in various diseases. CircRNAs can function as microRNA or RNA binding sites or can regulate mRNA. In this review, we explore the biogenesis and function of circRNAs in the context of the emerging role of circRNAs in periodontitis pathogenesis and the differentiation of periodontal cells. CircMAP3K11, circCDK8, circCDR1as, circ_0062491, and circ_0095812 are associated with pathological periodontitis tissues. Furthermore, circRNAs are expressed in periodontal cells in a cell-specific manner. They can function as microRNA sponges and can form circRNA–miRNA–mRNA networks during osteogenic differentiation for periodontal-tissue (or dental pulp)-derived progenitor cells.

## 1. Periodontal Disease and Regeneration 

### Periodontal Disease 

Periodontal diseases include gingivitis and periodontitis, both of which are widespread chronic inflammatory diseases [1,2]. Periodontitis occurs as a destructive host immune response to the accumulation of a bacterial biofilm (dental plaque) on the root surfaces of teeth. Inflammatory changes lead to progressive and episodic bursts of destruction of the supporting tissues around the teeth, which can lead to eventual tooth loss [3]. The global burden of periodontal disease increased by 57.3% from 1990 to 2010 [4]. Severe forms of periodontitis affect around 11% of adults across the globe [4,5]. At the global level, aggressive periodontitis (also called ‘juvenile periodontitis’) affects up to 1.6% of people who are younger than 30 years old, including children and adolescents [6]. 

**Figure 1:**
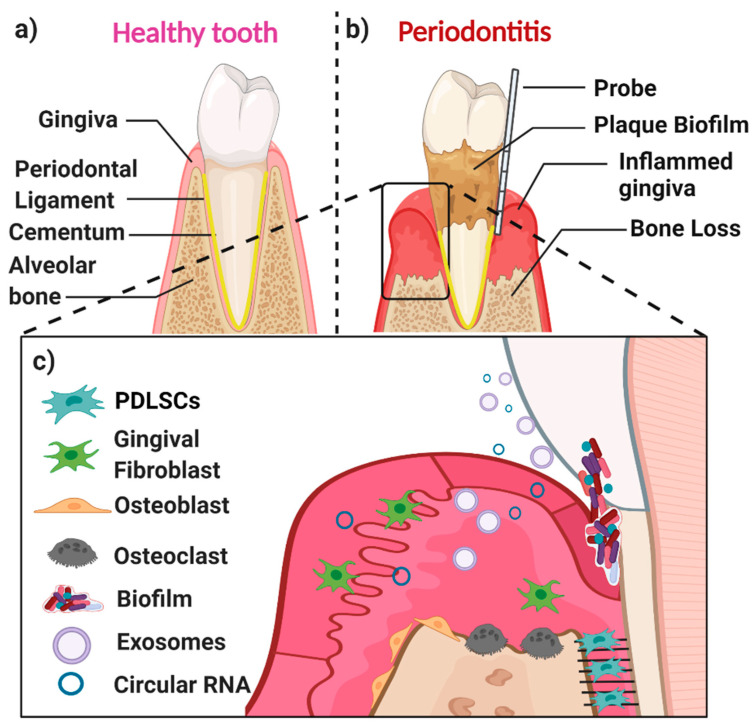
Schematic representation of the periodontium in health (**a**), or in periodontitis (**b**), and some selected key players in the periodontium (**c**). (**a**) The major components of a healthy periodontium are the gingiva, periodontal ligament (PDL), cementum, and alveolar bone. (**b**) Periodontitis is associated with the accumulation of dental plaque biofilm, gingival inflammation, and loss of both periodontal soft tissues (gingiva and PDL) and hard tissues (alveolar bone and cementum). (**c**) Key players in an inflamed periodontal site include PDLSCs, gingival fibroblasts, osteoblasts, osteoclasts, and different biological molecules (i.e., proteins, circular RNAs, exosomes, as well as inflammatory cytokines and regenerative growth factors).

A healthy periodontium consists of soft tissues (the gingiva and periodontal ligament) and hard tissues (cementum and alveolar bone) (Figure 1a). The accumulation of a microbial biofilm (dental plaque) at the interface of the tooth and the adjacent gingiva results in inflammation (gingivitis). Gingivitis is reversible once the plaque biofilm is disrupted and removed. In contrast, in susceptible patients, dental plaque induces irreversible inflammation of the underlying periodontal tissues, resulting in loss of attachment and resorption of bone. When inflammation extends deep into tissues, it results in extensive destruction of both soft and hard tissues, and this might ultimately cause loss of the affected tooth (Figure 1b,c) [7]. The likelihood of this happening is determined by both inherited factors such as cytokine gene polymorphisms and other immune response genes and inherited factors, the presence of conditions that modify the host’s response (such as diabetes mellitus and smoking), and local factors that predispose to the stagnation and maturation of the dental plaque biofilm (such as oral appliances and dentures) [8,9,10,11,12,13,14].

The main cell populations found within a healthy or diseased periodontium are periodontal ligament (stem) cells (PDLSCs), gingival fibroblasts (GFs), osteoblasts (OBs), osteoclasts, and various immune cells [15] (Figure 1c). An important biofluid in the context of the periodontium is gingival crevicular fluid (GCF), which is derived from serum. It flows passively from the gingival crevice, at the interface between the tooth and the adjacent gingiva. The composition of GCF alters according to the disease status of a particular periodontal site. The flow is greater when the site is inflamed. GCF can contain neutrophils as well as various cell-derived biological molecules, including small extracellular vesicles [16,17,18,19] and circular RNAs (circRNAs). These small molecules are also present in the periodontal tissues, where they play an essential role in periodontal tissue homeostasis, repair, and pathogenesis.

Susceptibility to periodontitis is influenced by both genetic and epigenetic factors, as well as by factors that modify the host immune response, such as smoking and uncontrolled diabetes mellitus [8,11,20]. Epigenetics is defined as a stably heritable phenotype resulting from chemical changes in a chromosome, without causing any changes in the DNA sequence, including DNA methylation, histone modification, and non-coding RNAs (ncRNA). Epigenetic-associated ncRNAs can be divided into two main groups—small ncRNAs (<30 nts) and long ncRNA (>200 nts). The potential role of epigenetics-related ncRNAs in diagnosing periodontitis was investigated previously in PDL and gingiva tissues [21,22,23,24]; however, most reviews of this topic focused mainly on the role of small non-coding RNAs (i.e., microRNA). The information gap in periodontology in terms of the significance of long non-coding RNA molecules (i.e., circular RNAs), both in the context of periodontal pathogenesis and regeneration, is addressed in this review.

## 2. circRNA Biogenesis and Function

### 2.1. What Are circRNAs?

CircRNAs are classified as long ncRNAs with over 200 nucleotides, without a 5′ cap or a 3′ polyadenylated (poly-A) tail [25]. Compared to their linear RNA counterparts, circRNAs are more stable. This is due to their covalent loop structure and the absence of the 5′ cap and 3′ poly-A tail. As a result, they are resistant to exonuclease, which is an RNase that can degrade linear RNAs.

In 1976, Sanger et al. found that a single-stranded circRNAs was the smallest pathogen that was able to infect certain plants [26]. These circular molecules were initially considered a product of a splicing error or gene rearrangement. Recently, cricRNAs have been shown to be conserved not only in prokaryotic life forms (archaea) but also in eukaryotes, across a variety of species. 

**Figure 2:**
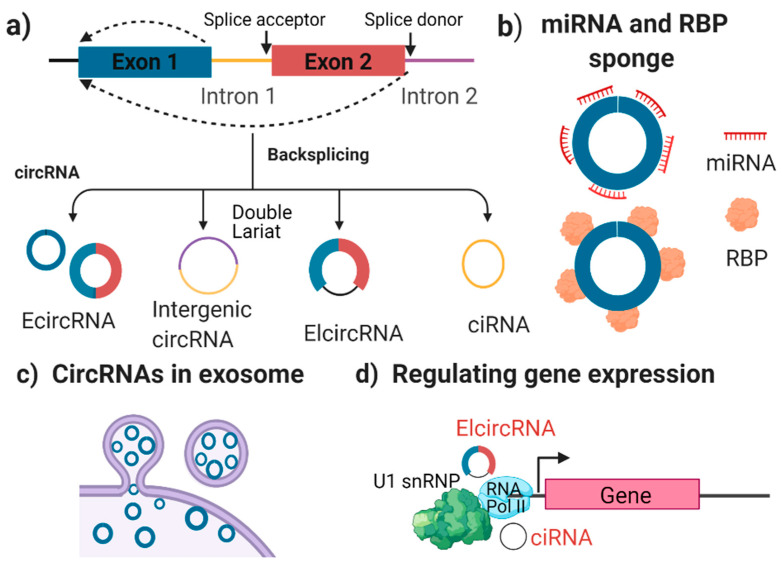
The biogenesis (**a**) and potential function (**b**–**d**) of circular RNAs. (**a**) Four types of circRNAs created via back splicing—ecircRNA, intergenic circRNA, elcircRNA, and ciRNA. (**b**) circRNAs can serve as sponges for binding miRNAs or RBPs. (**c**), exosome-derived circRNAs. )(**d**) aelcircRNA and ciRNA can regulate gene expression by binding with the RNA Pol II and U1 snRNP complex. RBP: RNA binding proteins; miRNA: microRNA; U1 snRNP: small nuclear ribonucleoprotein; and RNA pol II: RNA polymerase II.

Unlike other ncRNAs, circRNAs are located predominantly in the cytoplasm of eukaryotic cells. There are four types of circRNAs (Figure 2a) [27]—exonic circRNAs (ecRNAs), intergenic circRNAs, exon-intron circRNAs (eiciRNAs), and intronic circRNAs (ciRNAs). These have diverse structures that arise from various mechanisms of connecting exons and introns via back-splicing.

Exonic circRNAs (ecircRNAs or ecRNAs) are the most abundant of the four types. They contain one or more exons that can function as a sponge for miRNA or RBPs. An miRNA sponge means that the circRNAs can competitively bind to miRNAs and consequently repress their function. This type of circRNAs can be detected in cell-derived exosomes.

Intergenic circRNAs are generated from fragments of introns, and their function remains unknown at this time. Exon-intron circRNAs (elciRNAs) contains both exons and introns, and they regulate gene transcription. Finally, circular intronic RNAs (ciRNAs) are introns that contain a protein-coding gene in their open reading frame (ORF). They can assemble into a circular loop and their primary function is gene regulation.

### 2.2. The Characterisation and Function of circRNAs

At the molecular level, circRNAs are expressed at a higher level and are more stable than linear RNAs. This is because their circular structure resists enzymatic degradation, which gives circRNAs a longer half-life (24–48 h) than other linear transcripts (8–9 h) [28]. CircRNAs are particularly highly abundant in the human brain, as compared to other tissues [29]. Despite conservation in humans and in mice, the expression of circRNAs remains tissue-specific across species. They mainly compete with endogenous RNAs by binding to miRNA (i.e., acting as an miRNA sponge; Figure 2b). Additionally, circRNAs can interact with RNA binding proteins (RBPs) as a scaffold or decoy, and they can sequester proteins in a specific location [27]. 

Moreover, circRNAs are highly abundant and stable in small extracellular vesicles (also named exosomes) (Figure 2c). Exosomal circRNAs are associated with the development of a number of diseases [30]. Another function of circRNAs is controlling post-transcriptional gene expression and the cap-independent translation template. EIciRNA regulates gene expression by binding with RNA Pol II and the U1 snRNP complex (Figure 2d) [31]. Finally, some circRNAs are disease-specific, and are associated with chromosomal translocations and tumour growth, as well as with cell migration and differentiation.

Certain circRNAs that are abundant and stable in exosomes are critical players in regulating cancer growth, tumour angiogenesis, and invasion [32]. They also play important roles in other diseases, such as cardiovascular diseases [33].

### 2.3. Methods for Detecting circRNA

High-throughput RNA sequencing (RNA-seq) is a commonly used technique for investigating all four circRNAs and for understanding back splicing from pre-mRNA transcripts [34]. In addition to RNA-seq, several other techniques have been used to validate the expression of circRNAs, including real-time quantitative PCR (RT-qPCR), microarrays, northern blots, and bioinformatics [35].

A microarray provides high sensitivity and specificity for circRNAs, whereby an RNase enzyme is added to remove pre-mRNA and linear RNAs. A Northern blot is a rapid method of showing the entire structure of circRNAs [35]. Gel electrophoresis is used to confirm reverse transcript products that might contain errors, such as accidental gene duplications, template switching during cDNA replication, and splicing within two separate pre-mRNA [36]. Overall, RNA-seq and RT-qPCR have been the most commonly used techniques for studying circRNAs influences on gene expression.

However, various challenges of circRNA detection include uneven rRNA depletion, variable RNase treatment efficiency, a relatively low abundance level of circRNA (5–10% of linear RNAs), exclusion of sequencing errors and underestimation of back-spliced junction reads. Therefore, methods of detection and characterization of circRNA require more optimisation to overcome these challenges.

## 3. circRNA Expression in Periodontal Tissues

There is an emerging understanding of the potential role of circRNA in periodontitis, and in particular, disease pathogenesis. There have been three studies on their presence in PDL tissues [37,38,39] and two studies in gingival tissues [40,41] taken from periodontitis patients (Table 1).

A very recent study by Yu et al. [37] demonstrated that circMAP3K11 (also named hsa_circ_002284) was significantly increased in inflamed PDL tissues from periodontitis (*n* = 20), as compared to healthy PDL tissues (*n* = 10), using RT-qPCR. Periodontitis patients were selected based on the following criteria—(1) redness and swelling of the gingiva or bleeding on probing; (2) probing depth > 3 mm and attachment loss > 1 mm; and (3) radiographic evidence of horizontal or vertical resorption of alveolar bone. However, these criteria represent a low threshold for defining periodontitis, and the latest stage and grade guidelines for classifying periodontitis were not used. Furthermore, circMAP3K11 enhanced hPDLSCs proliferation and migration and reduced the apoptosis of hPDLSCs in vitro via the circMAP3K11/miR-511-3p/TLR4 axis. Overexpression of circMAP3K11 led to increased proliferation, migration, and osteogenic protein expression of Runx2, OSX, ATF4, and BSP in hPDLSCs.The dual-luciferase reporter assay demonstrated that miR-511-3p directly targeted circMAP3K11, and that toll-like receptor 4 (TLR4) is a direct target of miR-511-3p. Additionally, a mouse periodontitis disease model was generated in 24 female 8-week-old C57BL/6 J mice. After oral injection of hPDLSCs transfected with si-circMAP3K11 (1 × 10^4^ cells/mice) for 48 h, the si-circMAP3K11 group exhibited the highest apoptosis and decreased proliferating cells; indicating that circMAP3K11 promoted the proliferation and inhibited the apoptosis of hPDLSCs by acting as an miR-511-3p sponge in vivo.

Zheng et al. [38] revealed that hsa_circ_0003489 was significantly increased in PDL tissues from patients with periodontitis (*n* = 6) compared to a healthy control group. Hsa_circ_0003489 is located at the gene for cyclin-dependent kinase 8 (CDK8) and is also known as circCDK8. Six samples of either healthy or inflamed PDL tissues were collected. The inflamed samples were sourced from patients with mild or moderate chronic periodontitis. The healthy samples were collected from teeth extracted for orthodontic treatment or from erupted third molars. Using RT-qPCR, it was found that circCDK8 was expressed at more than two-fold higher levels in periodontitis compared with control tissues. circCDK8 was shown to inhibit the osteogenic differentiation of hPDLSCs (passage 3–5) under hypoxic conditions. Overexpression of circCDK8 leads to autophagy and apoptosis of PDLSCs via the mTOR signalling pathway. On the other hand, silencing of circCDK8 using small interfering RNA (si-circCDK8) enhances osteogenic differentiation in hPDLSCs through altered mTOR signalling [38].

Wang et al. [39] found that circRNA of cerebellar degeneration-related protein 1 transcript (circCDR1as) was significantly downregulated in PDL tissues from periodontitis patients. In this particular study, human PDL tissues were obtained from teeth with chronic periodontitis that was extracted because of advanced disease (*n* = 10), or from healthy teeth removed for orthodontic purposes (*n* = 11). Once again, using RT-qPCR, it was found that the expression level of CDR1as was significantly lower in PDL tissues from sites with periodontitis as compared to normal tissues. circCDR1as was also downregulated in lipopolysaccharide (LPS)-treated hPDLSCs. Knockdown of CDR1as (using siRNA-CDR1as) enhanced LPS-induced proliferative inhibition of hPDLSCs. In contrast, overexpression of CDR1as (by a pcDNA3.1-circ-mini-CDR1as plasmid) was found to promote the proliferation of hPDLSCs. Additionally, circCDR1as functions as a sponge for miR-7, and this is relevant to the extracellular-signal-regulated extracellular-signal-regulated kinase (ERK) or MAPK signalling pathway during the proliferation of hPDLSCs.

A recent study by Wang et al. [40] used RT-qPCR to show that circ_0081572 was significantly decreased in periodontitis-derived gingival tissues (*n* = 21) compared to healthy gingival tissues (*n* = 21). circ_0081572 was mainly distributed in the cytoplasm of human periodontal ligament cells (hPDLCs). Overexpression of circ_0081572 reversed the effects of lipopolysaccharide (LPS), which caused decreased viability and stimulated apoptosis and caspase 3 activity of PDLCs, by targeting miR-378h and retinoid acid-related orphan receptor A (RORA). This suggests that circ_0081572 inhibits the progression of periodontitis through regulation of the miR-378h/RORA axis.

Using RNA sequencing, Li et al. [41] found that a total of some 1,304 circRNAs were significantly expressed in the gingival tissues of periodontitis patients (*n* = 4). The authors selected six altered circRNAs and validated their presence using RT-qPCR. In total, in periodontitis gingival tissues, the levels of four circRNAs were increased (circ_0099630, circ_0138960, circ_0138959, and circ_0107783), while the level of the other two decreased (circ_0062490 and circ_0085289) when compared to healthy controls. In periodontitis tissues, circ_0062491 was significantly decreased, while circ_0095812 was significantly increased (*n* = 30) when compared to control tissues (*n* = 30). 

In summary, circMAP3K11, circCDK8, circCDR1as, circ_0081572, circ_0062491, and circ_0095812 are potentially associated with periodontitis; however, further studies are required to confirm these findings. The literature on the role of circRNAs in disease pathogenesis is limited, and this is an area that clearly requires further investigation.

## 4. The Emerging Role of circRNAs in Periodontal Regeneration

### 4.1. Periodontal Tissue Engineering

As discussed earlier, periodontitis destroys periodontal soft and hard tissues, and can result in tooth loss if left untreated. Where the ultimate goal of treatment is to fully restore tooth-supporting tissues, complete regeneration of functional periodontium (including the periodontal ligament, cementum, and alveolar bone) is the objective [42]. Regrettably, current treatment strategies cannot predictably achieve complete regeneration of the periodontium in most cases. Nevertheless, periodontal tissue engineering might provide an effective method for this purpose. Periodontal tissue engineering incorporates three main components—a cell source, scaffold, and biological cues [43,44,45,46,47,48,49,50,51,52]. Various cell sources, such as PDLSCs, GFs, dental pulp stem cells (DPSCs) [53], and bone marrow mesenchymal stem cells, have been proven to promote periodontal tissue regeneration both in vitro and in vivo [46]. However, cell-based approaches face several important challenges, including their high cost and the associated regulatory barriers. As a consequence, cell-free approaches that focus on scaffolds or bioactive molecules are being proposed, and here there is an emerging focus on circRNAs as potential regulators of cell function, to promote periodontal regeneration.

In addition to investigating the expression of circRNA in healthy and diseased periodontal tissues, the expression of circRNA expression has also been explored in various periodontal cells, such as human periodontal ligament stem cells (hPDLSCs), hDPSCs, hGFs, cell lines of mouse osteoblasts (OBs), and rat dental follicle cells (rDFCs). These studies have focussed on the role of circRNA in controlling cell differentiation, which is important in the context of periodontal regeneration and tissue engineering. Herein, we undertook a scoping review of recent research into the role of circRNA in cell differentiation in vitro and periodontal tissue healing and regeneration in vivo, drawing information from 23 studies [37,38,39,40,41,54,55,56,57,58,59,60,61,62,63,64,65,66,67,68,69,70,71]. Much of the existing research has focused on osteogenic differentiation of periodontal stem cells, and how circRNAs might function as a sponge for miRNAs.

### 4.2. The Role of circRNA in Human Periodontal Ligament Stem Cell Differentiation

A summary of nine studies of circRNAs exploring the topic of the osteogenic differentiation of hPDLSCs is shown in Table 2 and [37,38,39,40,54,55,56,57,58]. In these investigations, primary hPDLSCs were typically derived from the PDL tissue of a third molar tooth. Cells at passage 3–4 were used. In such cell culture models, there is accumulating evidence that circRNAs can modulate the osteogenic differentiation of hPDLSCs in vitro [38,39,40,54,55,57,58] and in vivo [37,56].

Xie et al. [54] demonstrated that 213 and 557 exosomal circRNAs could be detected after 5 and 7 days of osteogenic differentiation in hPDLSCs. Using RT-qPCR, they further confirmed that three 3 circRNAs (hsa_circ_0087960, hsa_circ_0000437, and hsa_circ_0000448) were upregulated after osteogenic differentiation, while hsa_circ_0000448 was downregulated. Analysis using the Kyoto Encyclopedia of Genes and Genomes (KEGG) predicted that those three upregulated circRNAs (i.e., hsa_circ_0087960, hsa_circ_0000437, and hsa_circ_0000448) might target 19 miRNAs, and in turn, those miRNAs can target transforming growth factor-beta (TGF-β), mitogen-activated protein kinases (MAPK), the mammalian Target of Rapamycin (mTOR), and the Forkhead box protein O1 (FOXO1) pathways. Of note, hsa_circ_0087960 is the product of the lysophosphatidic acid receptor 1 (LPAR1), a gene proven to modulate bone mineralization, cell metabolism, cytoskeletal components, and cell survival.

RNA sequencing data demonstrate that some 1191 circRNAs were upregulated, while 1487 were downregulated in hPDLSCs (passage 3), when the cells were subjected to a mechanical force (a 10% equibiaxial strain applied at a frequency of 1.0 Hz) [55]. Upregulated levels of circRNA3154, circRNA5034, circRNA3133, and circRNA5045 and downregulated levels of circRNA1818 and circRNA1358 were validated by RT-qPCR analysis. The Gene Ontology (GO) analysis identified that the possible functions of circRNAs are exerted through the circRNAs–miRNAs networks, e.g., circRNA3140 is associated with miR-21, while circRNA436 can act as a sponge for miR-107 and miR-335. Overall, changes in circRNAs correlate with osteogenic differentiation of hPDLSCS induced by mechanical force.

The role of CDR1as as a sponge for miRNA-7 in hPDLSCs was investigated in terms of influences on cell proliferation and osteogenic differentiation. Li et al [56] demonstrated that circCDR1as was upregulated during osteogenic differentiation of hPDLSCs (passage 4). This inhibited miR-7 and regulated both the TGF-β and MAPK pathways. Using RT-qPCR, the expression of CDR1as was found to be enhanced, while miR-7 levels decreased during osteogenesis by hPDLSCs. Western blot results showed that silencing CDR1as inhibited the phosphorylation of Smad1/5/8 and the p38 MAPK signalling pathway. Furthermore, in this study, siRNA-CDR1as transfected hPDLSCs were seeded on a polylactic-co-glycolic acid (PLGA) scaffold before being transplanted into a critical-sized mouse calvarial defect model. One month after transplantation, silencing CDR1as led to reduced bone formation, as documented by both microcomputed tomography (micro-CT) and conventional histology (hematoxylin and eosin staining) [56].

Another study using RNA sequencing revealed that a total of 12,693 circRNAs were detected in osteogenic lineage differentiated hPDLSCs (passage 4) [57]. In total, 118, 128, and 139 circRNAs were differentially expressed at days 3, 7, 14, of osteogenic differentiation, as compared to day 0. The bioinformatics analysis suggested that 50 circRNAs, 55 miRNAs, and 613 mRNAs formed a potential regulatory network during hPDLSCs osteogenesis.

Moreover, circRNAs might also regulate key mRNAs that are important in bone-formation-associated processes, including extracellular matrix organization and cell differentiation. They can also influence the bone morphogenic protein (BMP) signalling pathway. RT-qPCR studies showed increased levels of four circRNAs (circCRKL, circRIMS1, circMAN1A2, and circETFA) during hPDLSCs osteogenic differentiation over 14 days [57].

Gu et al. [58] demonstrated that 766 circRNAs were upregulated and 690 circRNAs were downregulated after 7 days of hPDLSCs osteogenic induction (passage 3). RT-qPCR studies validated the increased levels of CDR1as, circNCOA3, and circSKIL, and decreased the levels of circIFF01, circNTNG1, circPLOD2, circSMO, and circSMURF2 in osteogenic hPDLSCs. Based on the miRanda database, some 1382 circRNAs were predicted to bind with 855 miRNAs. It, therefore, appears that the circRNA–miRNA–mRNA network (i.e., circBANP-miR34a, circITCH-miR146, and MAPK pathway) is a major contributor to the functional changes caused by circRNA, during hPDLSCs osteogenesis [58].

In summary, 20 differentially expressed circRNAs were identified during hPDLSCs osteogenic differentiation, using either RNA-seq or RT-qPCR. These were predicted to function as a sponge for miRNA via BMP, wingless-related integration site (WNT), and MAPK pathways. However, only two studies were able to follow up this prediction in vivo using an animal model [37,56]. It is, therefore, necessary to design and undertake further in vivo experiments to confirm the predictions based on bioinformatics.

### 4.3. The Impact of circRNAs in the Differentiation of Human Dental Pulp Stem Cells (hDPSCs) 

There is increasing evidence that circRNAs can be detected in human dental pulp stem cells (hDPSCs) during their osteo/odontogenic differentiation [59,60,61,62,63,64,65] (Table 3). In these studies, hDPSCS were mostly isolated from dental pulp tissue removed from healthy or inflamed third molar teeth [64,65]. Most studies used hDPSCs at passage 3 for cell culture experiments [60,63,64].

Ji et al. demonstrated that circRNA124534 promoted in vitro and in vivo osteogenic differentiation of hDPSCs (passage 4) via the miRNA-496/β-catenin axis [59]. Increased circRNA124534 and decreased miR-496 were shown by RT-PCR at 3, 7, and 14 days during in vitro osteogenic differentiation of hDPSCs. Silencing circRNA124534 suppressed the osteogenic differentiation of hDPSCs in vitro, while overexpression of β-catenin reversed the inhibition. In an in vivo study, hDPSCs were transfected with lentivirus vectors to induce circRNA124534 overexpression, or the miR-496 mimic was used. The cells were seeded onto β-tricalcium phosphate scaffolds before being implanted subcutaneously into 6-week-old BALB/c nude mice (*n* = 6). After 8 weeks, overexpression of circRNA124534 was found to enhance bone formation, while the miR-496 mimic prevented bone formation.

In a study of the odontogenic differentiation of hDPSCs, Chen et al. [60] found that the levels of 43 circRNAs were upregulated, while 144 circRNAs were downregulated. RT-qPCR and microarray results were consistent, showing upregulated hsa_circRNA_104101, hsa_circRNA_406763, hsa_circRNA_002161, and hsa_circRNA_ 005044, and downregulated hsa_circRNA_079813 and hsa_circRNA_008336 in odontogenic differentiated hDPSCs. Bioinformatic analysis identified the involvement of the circRNA–miRNA–mRNA network, while the GO and KEGG pathway analyses predicted that the potential functional role of circRNA in hDPSCs odontogenic differentiation was exerted via the Wnt and TGF-β signalling pathways. The authors also generated an hsa_circRNA_104101 shRNA lentivirus vector. Transfection with sh-circ104101 led to decreased expression of alkaline phosphatase (ALP) and reduced formation of mineralized nodules, after 14 days in vitro differentiation of hDPSCs.

Studies using RT-qPCR showed that has_circ_0026827 was significantly increased in osteogenic differentiated hDPSCs [61]. Silencing has_circ_0026827 suppressed the osteoblastic differentiation of hDPSCs. Luciferase reporter assays confirmed that the target of hsa_circ_0026827 was miR-188-3p, whereas beclin-1 and runt-related transcription factor 1 (RUNX1) were the downstream targets of miR-188-3p. In an in vivo study, hDPSCs expressing sh-hsa_circ_0026827 and hsa_circ_0026827 were grown on Bio-Oss collagen scaffolds that were implanted subcutaneously in 5-week old BALB/c nude mice (*n* = 5 per group). After 8 weeks, overexpression of hsa_circ_0026827 promoted bone formation, while sh-hsa_circ_0026827 reduced bone formation.

Xie et al. [62] showed that exosomal hsa_circ_0003611 (also named circular lysophosphatidic acid receptor 1-circLPAR1) was increased during osteogenesis of hDPSCs after 14 days. Luciferase reporter assays demonstrated that circLPAR1 was the target of hsa-miR-31. Furthermore, hsa-miR-31 inhibitor and circLPAR1 overexpression were shown to promote osteogenic differentiation of hPDSCs. 

circSIPA1L1 (signal-induced proliferation-associated 1 like 1) circRNA was shown to be upregulated during hDPSCs (passage 1–3) osteogenic differentiation by binding to miR-617 [63]. PCR assays and Sanger sequencing confirmed the closed-loop structure of circSIPA1L1. circSIPA1L1 and miR-617 were shown to be expressed in the cytoplasm of hDPSCs using fluorescence in situ hybridization (FISH). Furthermore, in an in vivo study, si-circSIPA1L-1 and si-circSIPA1L1-3 transfected hDPSCs were loaded on Bio-Oss Collagen scaffolds and implanted subcutaneously in nude mice for 8 weeks. Reduced formation of bone-like structures and collagen deposits was found in both si-circSIPA1L-1 and si-circSIPA1L-1 groups, as compared to the control group. They concluded that the circSIPA1L1/miR-617/Smad3 axis might be responsible for the osteogenic differentiation of hDPSCs [63].

Using a microarray, Zhang et al. [64] found a total of 57 upregulated and 29 downregulated circRNAs during osteogenesis by hDPSCs (passage 3–5). Knockdown of circAKT3 (AKT3 - AKT Serine/Threonine Kinase 3) inhibited osteogenic differentiation. A dual-luciferase reporter assay showed that circAKT3 could directly bind to miR-206, while connexin 43 (CX43) was a target of miR-206. In an in vivo study, sh-circAKT3 treated hPDSCs were mixed with beta-tricalcium phosphate (β-TCP) scaffolds and implanted subcutaneously into 6-week-old BALB/c nude mice (*n* = 4). After 8 weeks, the cells with sh-circAKT3 showed reduced bone formation compared to the controls [64].

A study by Li et al. [65] that used RNA sequencing, showed that some 1341 circRNAs were increased, and 1780 were decreased in human dental pulp cells (hDPCs, passage 3) after 14 days of odontogenic differentiation. Has_circ_0015260 and has_circ_0006984 were upregulated in odontogenic differentiated hDPSCs, as was seen from RNA-seq and RT-qPCR results. The functional analysis predicted that these two circRNAs contain binding sites of miR-135b. The GO and KEGG analysis predicted that the targets of these circRNAs were miRNAs and genes that were involved with the MAPK signalling pathway in the odontogenic differentiation of hDPSCs [65]. 

In summary, seven studies showed that circRNAs could be detected in hDPSCs isolated from healthy dental pulp tissues. One study detected exosomal circRNA [62]. There are a total of eight circRNAs that are known to be involved in the osteogenesis of hDPSCs, which function as sponges for miRNA. There are four in vivo studies thatvalidated the function of circRNAs in dental pulp stem cells [59,61,63,64]. Future in vivo studies are required to confirm the functional roles of circRNAs during the differentiation of hDPSCs.

### 4.4. circRNAs in MC3T3-E1, Rat Dental Follicle Cells (rDFCs), and Gingival Fibroblasts

The MC3T3-E1 mouse osteoblastic cell line is commonly used to study bone formation in vitro. A total of four studies investigated the role of circRNAs in this cell line [66,67,68,69], rat dental follicle cells (rDFCs) [70], and gingival fibroblasts cell line [71], as shown in Table 4.

Cao et al. [66] utilized RNA-seq and found that 232 circRNAs were increased and 95 circRNAs were decreased during osteogenic differentiation when the cells were maintained under microgravity conditions using a rotary cell culture system. Upregulated levels of circ_014154 and downregulated levels of circ_014977, circ_001790, circ_010383, circ_014393, and circ_017642 were validated by RT-qPCR analysis in MC3T3-E1 cells that were maintained in osteogenic media for 72 h. Functional analysis indicated that miR-145a-5p and let-7a-5p were the downstream targets of circ_014154. Analysis of the circRNA-mRNA network suggested that circ_014154 was closely correlated with Calm2 (Calmodulin 2), Col1a1 (Collagen Type I Alpha 1 Chain), and ACTN1 (Actinin Alpha 1) during osteogenic differentiation of MC3T3-E1 cells exposed to microgravity.

In MC3T3‑E1 cells, another circRNA of interest is mmu_circ_003795 [67]. Levels of this circRNA increased after 72-h of osteogenic differentiation of MC3T3-E1 cells, as well as MDPC23 cells. GO analysis predicted that miR1249-5P is the target of mmu_circ_003795. Silencing of mmu_circ_003795 in MC3T3‑E 1 and MDPC23 cells were found to reduce the expression of osteogenic markers (collagen alpha-1(XV) and osteopontin), while the expression level of mmu‑miR‑1249‑5p was upregulated.

Cao et al. [68] demonstrated that exosomes derived from circ-Rtn4 (circ-reticulon 4)- treated bone marrow mesenchymal stromal cells (BMSCs) (Rtn4-Exos) inhibited cytotoxicity and apoptosis in MC3T3-E1 cells caused by tumor necrosis factor-alpha (TNF-α) and were mediated by miR-146a. In keeping with this, miR-146a was identified as a target of circ-Rtn4. Thus, exosomes derived from circ-Rtn4 exerted their function in TNF-α-treated MC3T3-E1 cells, by sponging miR-146a. 

Mi et al. reported that circRNA AFF4 (circ-ALL1-fused gene from chromosome 4/FMR2 Family Member 4) functions as an miR-7223-5p sponge. This promoted in vitro osteogenic differentiation of MC3T3-E1 and accelerated the healing of bony fractures in vivo [69]. The Luciferase reporter assay showed that the expression of WT circRNA AFF4 was suppressed by miR-7223-5p treatment, while circRNA AFF4 mutations abolished the inhibitory effect of miR-7223-5p. miR-7223-5p targets PIK3R1 (phosphatidylinositol 3-kinase regulatory subunit alpha). CircRNA AFF4 was found to be significantly increased during the early stages of the healing of bony fractures in vivo (i.e., at 3, 7, and 10 days). When circRNA AFF4 and si-circRNA AFF4 were locally injected in sites of femoral transverse osteotomy of C57BL/6 mice for 4 and 7 days, it was found that circRNA AFF4 injection promoted bone formation in vivo [69].

Du et al. [70] demonstrated that a total of 138 upregulated and 128 downregulated circRNAs were present in rat dental follicle cells (rDFCs, passage 3–4) after the cells had undergone 28 days of osteogenic differentiation. RT-qPCR data showed upregulated circFgfr2 (circ-fibroblast growth factor receptor 2) and downregulated circ-Tgfbr2 (circ-transforming growth factor-beta receptor 2) at 1, 2, 3, and 4 weeks after osteogenic induction. Moreover, in situ hybridization results showed that circFgfr2 expression was enhanced in dental follicle tissues from day 1 to 11 postnatally in rats, while miR-133 was downregulated. Overexpression of circFgfr2 led to decreased miR-133 and increased BMP6 (Bone Morphogenetic Protein 6) expression.

Circ-Amotl1 (angiomotin-like 1) was found to accelerate skin wound healing in a mouse excisional wound model. It achieved this by binding to nuclear Stat3 (signal transducer and activator of transcription 3), enhancing the Dnmt3a (DNA methyltransferase 3 alpha) promoter, and modulating miR-17 [71]. To demonstrate this in vivo, a full-thickness cervical dermal excisional wound model was created in C57BL/6xCBA mice. A mixture of circ-Amotl1 plasmids and gold nanoparticles were injected into the defect. The circ-Amotl1 accelerated the skin wound healing process, with increased Stat3 and Dnmt3a, and enhanced blood vessel formation and cell migration. The expression level of circ-Amotl1 was enhanced, which in turn promoted cell migration, adhesion, proliferation, and survival in human gingival fibroblasts. This effect was shown in the CRL-2014 (ATCC cell line) and NIH 3T3 fibroblast cell lines. Knockdown of circ-Amotl1 by small interfering RNA (siRNA) inhibited cell migration and proliferation. Moreover, a decreased expression of miR-17-5p caused by ectopic expression of circ-Amotl1 was also found in this study.

It could therefore be concluded that circRNAs play important roles in the osteogenic differentiation of a range of pre-osteoblastic cells and other dental cells, through the circRNA-miRNA-mRNA network.

## 5. Conclusions and Future Directions 

A variety of periodontal and other oral tissue-related cells, such as hPDLSCs, hDPSCs, MC3T3, and gingival fibroblasts, have been shown to be able to promote periodontal tissue regeneration, both in vitro and in vivo [43,45,46,52,72]. In addition to these cells, a range of exogenous proteins, exosomes, and circRNAs that were released from periodontal cells could also play a crucial role in periodontal tissue function, contributing to either homeostasis and regeneration, or tissue loss during periodontitis.

In terms of circRNA expression in diseased tissues and a potential role in pathogenesis, it was found that several circRNAs, including circCDK8, circCDR1as, circ_0081572, circ_0062491, and circ_0095812, were differentially expressed in PDL or gingival tissues from sites with periodontitis. This suggests that these five circRNAs are potentially associated with periodontitis. However, there is very limited data on the expression of circRNAs in periodontal tissues, and hence more studies are required in this area.

The majority of the studies investigating the role of circRNAs in the field of periodontology focussed on their effect on cell differentiation in the context of wound healing and regeneration. Currently, our understanding of the role of circRNAs in cellular differentiation and tissue remodelling is still in its infancy. Multiple groups of researchers have detected circRNAs in periodontal tissues [37,38,39,40,41], as well as in periodontal cells [37,38,39,40,41,54,55,56,57,58,59,60,61,62,63,64,65,66,67,68,69,70,71] and exosomes derived from periodontal cells [54,62]. Most studies included in this review were focused on the role of circRNAs in the osteogenic differentiation of periodontal cells. This provides insight into only one part of their overall function. Future investigations need to explore the influence of circRNA in adipogenic, neurogenic, myogenic, and ligament differentiation. It is likely that their influence extends into all of these areas since findings from in vivo animal models suggest that they can contribute to a range of processes involved in tissue regeneration.

Indeed, among the 23 studies in this review, a total of 8 studies used an *i*n vivo model to investigate the function of circRNAs [37,56,59,61,63,64,69,71]. Of these seven studies conducted with mice, three used a subcutaneous skin model [59,61,66], with just one study using a calvarial bone defect [56], a periodontitis disease model [37], a femur fracture model [69], and a skin wound healing model [71]. In these studies, circRNAs was administered using either direct local injection [69] or oral injection [37], employing different biomaterials as delivery vehicles for cells, such as scaffolds made of PLGA [56], β-TCP [59,64], and Bio-Oss collagen [61,63], while one study used gold nanoparticles [71]. To explore the potential of circRNAs in the regeneration of anatomically complex tissues like the periodontium, more pre-clinical models and circRNAs delivery systems need to be investigated.

**Figure 3:**
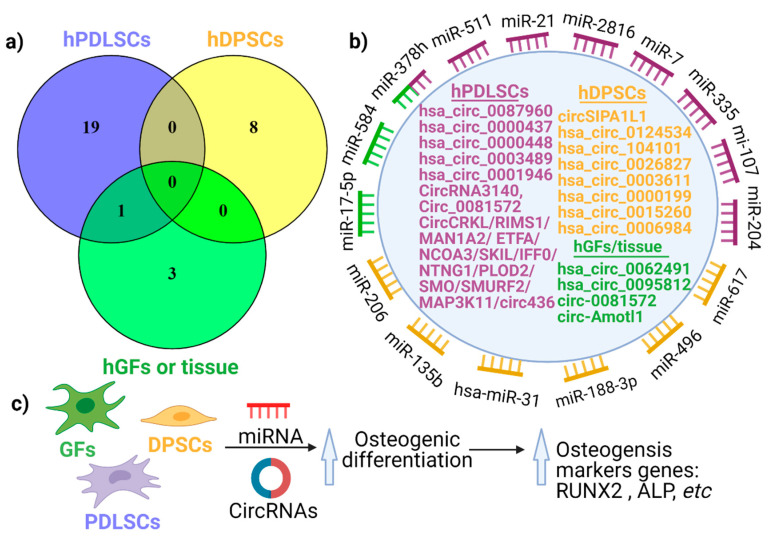
Cell-specific expression of circRNAs (**a**), the potential role of circRNAs as a miRNA sponge (**b**) and the circRNA-miRNA network (**c**) during osteogenic differentiation. (**a**) A Venn diagram showing the expression pattern of circRNAs in hPDLSCs, hDPSCs and hGFs (or tissue). (**b**) The function of circRNAs when they act as miRNAs sponges. (**c**) The mechanism of circRNAs in osteogenic differentiation for hPDLSCs, hGFs, and hDPSCs exerted via a circRNA-miRNA network.

After analysing all 31 investigations of circRNAs, and expressing their interactions using a Venn diagram (Figure 3a), it appears that there is no commonly shared circRNA between the different cells types (hPDLSCs, hDPSCs, hGFs). However, circ_0081572 was found in both gingival tissues (not cells) and hPDLCs [40]. This indicates that circRNAs might be cell-specific in the periodontium. For instance, circRNA CDR1as was identified in hPDLSCs from three studies, not in other oral cells. Nevertheless, additional studies on the topic of cell distribution are needed to confirm this concept of cell-specific expression within the periodontium. Figure 3b outlines the potential network between circRNAs and miRNAs, where circRNAs act as a sponge for miRNAs.

Notably, not all circRNAs promote osteogenesis of hPDLSCs, with some being downregulated during osteogenesis. Figure 3c describes the mechanism through which circRNAs can promote osteogenic differentiation in hPDLSCs, hGFs, and hDPSCs. The circRNA–miRNA interaction is important during osteogenic differentiation with an inhibitory mechanism, where circRNAs inhibits miRNAs expression, as shown by the gain-of-function or loss-of-function experiments. This suggests that circRNAs are able to modulate several critical biological pathways within cells of the periodontium.

It has been shown that circRNAs are involved with age-related diseases (i.e., cancer, diabetes, osteoporosis, and rheumatoid arthritis [73]) and are expressed in a development stage-specific manner. It is reasonable to expect that circRNAs involved in periodontal cell differentiation and periodontal regeneration are influenced by ageing and by the stage of development of the periodontium. However, this is still an uncharted area that requires more in depth exploration to better understand the role of circRNAs during the periodontium formation and in periodontal regenerative medicine.

The circRNA–miRNA–mRNA regulatory network was reported in diabetic foot ulcers [74], osteosarcoma [75], and various cancers [76], and it has now been described as the most important molecular mechanism of circRNAs in periodontal cells. However, there remains a need to better understand the full range of potential mechanisms that can underlie the activity of circRNAs.

Given the ability of certain circRNAs to alter the behavior of cells, their use in a therapeutic framework to regulate cell differentiation to achieve optimal periodontal regeneration is an exciting prospect. Further studies on circRNAs are needed to completely understand how they could be used both in periodontal diagnosis as well as in periodontal regeneration.

## Figures and Tables

**Table 1:** Expression of circRNAs in PDL (and cells) and gingival tissues.

Reference	circRNA Name (Gene Name), Genome Location and Length	circRNA Target or Pathway	CircRNA Detection Method	Cell (Tissue) Details	Key Findings
Yu et al. 2021 [37]	circMAP3K11 (MAP3K11)	miR-511	RT-qPCR10	PDL tissues from 10 healthy and 20 periodontitis cases. hPDLSCs from healthy donors. Donor age—unclear. Passage 3.	Higher expression levels of circMAP3K11 and TLR4, and lower expression levels of miR-511-3p were found in periodontitis affected PDL tissues, compared to healthy controls. circMAP3K11 enhanced hPDLSCs proliferation, migration and osteogenic differentiation, and reduced the apoptosis of hPDLSCs in vitro through a circMAP3K11/ miR-511-3p/TLR4 axis. *In vivo*—silencing circMAP3K11 can prevent periodontitis development in mice, with decreased cell proliferation and increased apoptosis.
Zheng et al. 2021. [38]	hsa_circ_0003489 (circCDK8) chr13:26974589-26975761; 1172 bp	mTOR signalling pathway	RT-qPCR	PDL tissues from 6 healthy and 6 mild/moderate chronic periodontitis patients hPDLSCs; Healthy PDL tissues (third molars); Passage 3 to 5.	CircCDK8 and HIF-1α were increased in PDL tissues from periodontitis patients. Overexpression of CircCDK8 decreased osteogenic differentiation of hPDLSCs through the mTOR pathway under hypoxia.
Wang et al. 2019. [39]	hsa_circ_0001946; CDR1as (or CiRS-7) chrX:139865339-139866824; 1485 bp	miR-7 ERK/MAPK signal pathway	RT-qPCR	PDL tissue from 10 periodontitis and 11 healthy cases. hPDLSCs from healthy teeth and chronic periodontitis tissue; passage 3. Donor age 30–40 yrs.	circCDR1as was significantly downregulated in PDL tissues from periodontitis patients. circRNA CDR1as inhibited hPDLSCs proliferation through the miR-7 and ERK/MAPK pathway
Wang et al. 2021. [40]	circ_0081572 (GRHL2) chr8:102564942-102571040; 6098 bp	miR-378h	RT-qPCR	Gingival tissues from 21 healthy and 21 periodontitis cases, Human periodontal ligament cells (hPDLCs). Unclear donor age and cell passage number.	Circ_0081572 was downregulated in the gingival tissues of periodontitis, compared to healthy gingival tissues. Overexpression of circ_0081572 could alleviate LPS-induced PDLCs injury via circ_0081572/miR-378h/RORA axis.
Li et al. 2019. [41]	hsa_circ_0062491 chr22:23063339-23180200; 116861 bp	miR-584	RNA sequencing RT-qPCR	Gingival tissues from 4 healthy and 4 chronic periodontitis cases. THP1 cells treated with *P. gingivalis.*	1, 304 circRNAs were significantly differentially expressed in the gingival tissues of periodontitis patients (*n* = 4). Decreased circ_0062491 and increased circ_0095812 found in periodontitis-gingival tissues compared to healthy tissues (*n* = 30) using RT-qPCR. Circ_0062491 function as a miR-584 sponge in THP1 cells.

Abbreviations: MAP3K11: Mitogen-activated protein kinase kinase kinase 11; CDK8: Cyclin-Dependent Kinase 8; CDR1as: Cerebellar degeneration-related protein 1 antisense RNA;GRHL2: grainyhead like transcription factor 2; TLR4: Toll-like receptor 4; HIF-1α: Hypoxia-inducible factor 1-alpha; mTOR: The mechanistic target of rapamycin; ERK: extracellular-signal-regulated kinase; RORA: RAR-related orphan receptor alpha; and THP1: human acute monocytic leukemia cell line.

**Table 2:** Expression of circRNAs in human periodontal ligament stem cells (hPDLSCs).

Reference	circRNA Name (Gene Name), Genome Location and Length	circRNA Target or Pathway	CircRNA Detection Method	Cell (Tissue) Details	Key Findings
Xie et al. 2021. [54]	hsa_circ_0087960 (LPAR1) chr9:113734352-113735838; 1486 bp hsa_circ_0000437 (CORO1C) chr12:109046047-109048186; 2139 bp hsa_circ_0000448 (GCN1L1) chr12:120592773-120593523; 750 bp	miRNA TGF/β, MAPK, mTOR, and FOXO1 pathways	RNA sequencing RT-qPCR	Human Periodontal Ligament Stem Cells (hPDLSCs) from third molar tissue; Donor ages 18–30 yrs; Passage 3.	69–557 exosomal circRNAs were detected after 5 and 7 days of osteogenic differentiation of hPDLSCs. Exosomal circRNA-LPAR1 was increased and has_circ_0000448 was decreased in hPDLSCs after 5 and 7-days osteogenesis. Function as miRNA sponge and modulate TGF- β, MAPK, mTOR, and FOXO1 pathways.
Wang et al. 2018. [55]	CircRNA3140 CircRNA436	miR-21 miRNA-107 miRNA-335	RT-qPCR RNA sequencing	hPDLSCs; Middle third root; Healthy PDL cells culture; age 14–16 passage 3	1191 cricrRNAs in hPDLSC were enhanced by mechanical force-induced osteogenic differentiation. Potential functions of circRNAs through circRNAs–miRNAs networks. For instance, circRNA3140 targets miR-21; circRNA436 targets miRNA-107 and miRNA-335.
Li et al. 2018. [56]	CDR1as hsa_circ_0001946; chrX:139865339-139866824; 1485 bp	miR-7; TGF-β/Smad and MAPK pathway	RT-qPCR	hPDLSCs PDL tissue from healthy premolars; Passage 4	CircRNA CDR1as inhibits osteogenic differentiation of hPDLSCs via inhibiting miR-7, TGF-β/Smad and MAPK pathways; In vivo knockdown of CDR1as reduced bone formation in a mouse calvarial defect model.
Zheng et al. 2017. [57]	CircCRKL, CircRIMS1, CircMAN1A2, CircETFA	miRNA sponge	RT-qPCR RNA sequencing	hPDLSCs (*n* = 3) PDL tissue from a healthy premolar; Donor age: 12–18; Passage 4	12,693 circRNA transcripts were detected in hPDCSc osteogenic differentiation with a time-specific expression. Four circRNAs were increased in hPDLSCs osteogenesis by RNA-seq and RT-qPCR; circRNA-miRNA-mRNA network is the potential regulatory role of circRNA.
Gu et al. 2017. [58]	Upregulated: CDR1as, circNCOA3 and circSKIL; Downregulated: circIFF01, circNTNG1, circPLOD2, circSMO and circSMURF2	miRNA34a and miRNA146a; MAPK and Wnt pathway	RT-qPCR RNA sequencing	hPDLSCs Donor age:18–20, Passage 3	1456 circRNAs were differentially expressed after 7-day osteogenic differentiated hPDLSCs. CDR1as, circNCOA3 and circSKIL upregulated during osteogenesis of hPDLSCs; CircRNA-miRNA-mRNA network is the potential function of circRNA in hPDLSCs osteogenic differentiation. For instance, circRNA BANP and circRNA ITCH were predicted to interact with miRNA34a and miRNA146a to regulate PDLSC osteogenic differentiation via the MAPK pathway.

Abbreviations: LPAR1: lysophosphatidic acid receptor 1; CORO1C: coronin 1C; GCN1L1: general control of amino-acid synthesis 1-like 1; CDK8: Cyclin-Dependent Kinase 8; CDR1as: Cerebellar degeneration-related protein 1 antisense RNA; CRKL: CRK Like Proto-Oncogene, Adaptor Protein; RIMS1: Regulating Synaptic Membrane Exocytosis Protein 1; MAN1A2: Mannosyl-oligosaccharide 1; ETFA: Electron Transfer Flavoprotein Subunit Alpha; NCOA3: Nuclear Receptor Coactivator 3; SKIL: The human SKI-like; IFF01: Intermediate Filament Family Orphan 1; NTNG1: Netrin G1; PLOD2: Procollagen-Lysine,2-Oxoglutarate 5-Dioxygenase 2; SMO: Smoothened; SMURF2: SMAD Specific E3 Ubiquitin Protein Ligase 2; BANP: B-cell translocation gene 3 associated nuclear protein; ITCH: Itchy E3 Ubiquitin Protein Ligase; TGF-β: Transforming Growth Factor Beta; MAPK: Mitogen-activated protein kinases; mTOR: mammalian Target of Rapamycin; FOXO1: Forkhead box protein O1; Smad: homologues of the Drosophila protein, mothers against decapentaplegic (Mad) and the Caenorhabditis elegans protein Sma; and Wnt: Wingless-related integration site.

**Table 3:** Role of circRNAs expression in the differentiation of human dental pulp stem cells (hDPSCs).

Reference	circRNA Name (Gene Name), Genome Location and Length	circRN Target or Pathway	CircRNA Detection Method	Cell (Tissue) Details	Key Findings
Ji et al. 2020. [59]	circRNA124534/ hsa_circ_0124534 (FRMD4B) chr3:69247848-69265490; 17,642 bp	As a miRNA sponge; miR-496/β-catenin pathway	RT-PCR	hDPSCs healthy pulp tissues (3 male, 3 female); Donor age: females: 22–33; males: 26–41 passage 4	CircRNA124534 enhanced in vitro osteogenic differentiation in hDPSCs via the miR-496/β-catenin pathway. Over-expression of circRNA124534 in vivo increased bone formation in a mouse subcutaneous model.
Chen et al. 2020. [60]	hsa_circRNA_104101	Wnt and the TGF-β signalling pathway	RT-qPCR Microarray Electro-phoresis	hDPSCs donor age: 18–25 yrs; passages 3–5	43 upregulated and 144 downregulated circRNAs were detected in hDPSCs during odontogenic differentiation. hsa_circRNA_104101 promoted hDPSCs odontogenic differentiation.
Ji et al. 2020. [61]	hsa_circ_0026827 (RPL41); chr12:56510373-56511616; 1243 bp	miR-188-3p; Beclin1& RUNX1 pathway	Microarray RT-qPCR	hDPSCs	has_circ_0026827 was upregulated during osteogenic differentiation in hDPSCs. has_circ_0026827 targets the miR-188-3p via Beclin1 & RUNX1 pathway. Overexpression of has_circ_0026827 promoted in vivo bone formation. circRNA–miRNA–mRNA networks may operate during odontogenic differentiation in hDPSCs via the Wnt and TGF-β signalling pathways.
Xie et al. 2020. [62]	circLPAR1 (hsa_circ_0003611) chr9:113703700-113735838; 32,138 bp	hsa-miR-31 SATB2 and RUNX2	RNA-seq RT-PCR	hDPSCs (Exosomes) from one healthy donor (age: 20 yrs); Passage 2	Exosomal crcLPAR1 enhanced osteogenic differentiation of hDPSCs by binding to has-miR-31.
Ge et al. 2020. [63]	circSIPA1L1	miR-617 Smad3	RT-PCR	hDPSCs Third molar from a healthy donor aged 18–25 yrs; Passage is unclear	CircSIPA1L1 promoted osteogenesis via regulating the miR-617 and Smad3 pathway in hDPSCs.
Zhang et al. 2020. [64]	circAKT3 (hsa_circ_0000199) chr1:243708811-243736350; 27,539 bp	miR-206; CX34	qPT-PCR RNA sequencing	hDPSCs premolars and third molars; age:14–25; passages 3 to 5	29 circRNAs were down-regulated and 57 circRNAs were upregulated during hDPSCs osteogenesis. CircAKT3 promoted osteogenesis in hDPSCs by binding to miR-206. In vivo—silencing circAKT3 inhibited bone formation in a mouse subcutaneous model.
Li et al. 2019. [65]	hsa_circ_0015260 (C1orf9), chr1:172520652-172548407; 27,755 bp hsa_circ_0006984 (ZNF79) chr9:130206308-130207528; 1220 bp	miR-135b; MAPK pathway	RT-qPCR RNA-seq	Human dental pulp cells (hDPCs) from healthy premolars and third molars (3 males and 5 females; 12–25 yrs); Passage 3	1341 increased circRNAs and 1780 decreased circRNAs were identified in hDPCs during odontogenic differentiation. Has_circ_0015260 and has_circ_0006984 were up-regulated during osteogenesis of hDPCs via miR-135b and the MAPK pathway.

Abbreviations: FRMD4B: FERM Domain Containing 4B; RPL41: Ribosomal Protein L41; LPAR1: Lysophosphatidic Acid Receptor 1; SIPA1L1: Signal Induced Proliferation Associated 1 Like 1; AKT3: AKT Serine/Threonine Kinase 3; AKT Serine/Threonine Kinase 3: Chromosome 1 open reading frame 9; ZNF79 Zinc Finger Protein 79; CX43: Connexion 43; TGF-β: Transforming growth factor beta 1; RUNX1: runt-related transcription factor 1; SATB2: Special AT-rich sequence-binding protein 2; RUNX2: Runt-related transcription factor 2; Smad3: SMAD family member 3; and MAPK: Mitogen-activated protein kinase.

**Table 4:** CircRNAs in MC3T3-E1 cells, rDFCs, and GFs cell lines.

Reference	circRNA Name (Gene Name), Genome Location and Length	circRNA Target or Pathway	CircRNA Detection Method	Cell (Tissue) Details	Key Findings
Cao et al. 2021. [66]	circ_014154	miR-145a-5p and let-7a-5p; MAPK pathway	RT-qPCR RNA-seq	MC3T3-E1	232 upregulated and 95 down-regulated circRNAs were found during osteogenic differentiation of MC3T3-E1 cells under microgravity; Circ_014154 was upregulated in MC3T3-E1 cells with osteogenic differentiation induced by microgravity via miR-145a-5p, let-7a-5p and the MAPK pathway.
Wu et al. 2020. [67]	mmu_circ_003795	mmu‑miR‑1249‑5p COL15A1	RT-qPCR nucleic acid electro-phoresis Micro-array	MC3T3‑E1 and MDPC23 cells; 24–72 h after transfection	mmu_circ_003795 was increased after 72 h osteogensis in MC3T3-E1 and MDPC23 cells by RT-qPCR via mmu‑miR‑ 1249‑5p by targeting COL 15A1.
Cao et al. 2020. [68]	circ-Rtn4	miR-146a	RT-qPCR Luciferase reporter assay	exosomes from circRtn4-modified BMSCs MC3T3-E1	circRtn4 as miR-146a sponge to regulate exosomes from BMSCs reduced TNF-α induced cytotoxicity and inhibited apoptosis of MC3T3-E1. Luciferase reporter assay validated the binding between circRtn4 and mi-146a.
Mi et al. 2019. [69]	CircRNA AFF4 Mmu_circ_0000262 chr11:53182161-53194174; 12,013 bp	miR-7223-5p; (PIK3R1)	RT-qPCR Luciferase reporter assay; micro-CT	MC3T3-E1	*In vitro*—CircRNA AFF4 stimulated MC3T3-E1 proliferation and inhibited apoptosis via binding to miR-7223-5p, and its downstream target is PIK3R1. *In vivo*—circRNA AFF4 enhanced fracture healing in a mouse femur fracture model.
Du et al. 2019. [70]	circFgfr2	miR-133 and BMP6 (bone morphogenetic protein-6); MAPK, TGF-β	RT-qPCR RNA-seq	Rat dental follicle cells (rDFCs) and tissues; passage 3 to 4.	CircFgfr2 promotes osteogenic differentiation of rDFCs via miR-133/BMP6. In situ hybridization identified that circFgfr2 was upregulated in mandible dental follicle tissues at days 1 to 11 postnatally in Sprague-Dawley rats, while miR-133 was decreased.
Yang et al. 2017. [71]	circ-Amotl1	miR-17-5p Stat3, Dnmt3a and fibronectin	RT-qPCR	Human gingival fibroblast cell line CRL-2014; NIH 3T3 fibroblast cell line.	Circular-Amolt promoted in vivo skin wound healing. Circular-Amolt enhanced hGFs and NIH 3T3 cell migration; Circular-Amolt enhanced STAT3, Dnmt3a and fibronectin while suppressing the expression of miR-17-5p.

Abbreviations: Rtn4: Reticulon 4; AFF4: ALL1-fused Gene from Chromosome 4/FMR2 Family Member 4; Fgfr2: Fibroblast growth factor receptor 2; Tgfbr2: Transforming Growth Factor Beta Receptor 2; MDPC23: Mouse Dental Papilla Cell; BMSCs: Bone marrow stromal cells; PIK3R1: Phosphoinositide-3-Kinase; COL15A1: Collagen alpha-1(XV); OPN: Osteopontin; MAPK: mitogen-activated protein kinases; TGF- β: transforming growth factor-beta; TNF-α: tumor necrosis factor alpha; B mircoCT: microcomputed tomography BMP6: Bone Morphogenetic Protein 6; MIR650: microRNA 650; LRRC4C: Leucine Rich Repeat Containing 4C; Amotl1: Angiomotin-like 1; STAT3: Signal transducer and activator of transcription 3; Dnmt3a: DNA Methyltransferase 3 Alpha; and THP1: human acute monocytic leukemia cell line.
